# Therapeutic effects and prognostic factors of ^125^I brachytherapy for pelvic recurrence after early cervical cancer surgery

**DOI:** 10.1038/s41598-021-90007-x

**Published:** 2021-05-31

**Authors:** Rui Wang, Jinhu Zhu, Shu Yang, Xiaoqin Chen, Cairu Gu, Tong Liang, Ling Li, Dan Liu, Yanqing Cao

**Affiliations:** 1 Department of Gynecology, GuangZhou Red Cross Hospital, Jinan University, Guangzhou, 510220 Guangdong China; 2 Department of Oncology, The First Affiliated Hospital of Guangdong Pharmaceutical University, Guangzhou, 510080 Guangdong China

**Keywords:** Cancer therapy, Cervical cancer

## Abstract

To investigate the efficacy of ^125^I seed implantation in the treatment regimen of pelvic recurrence after early cervical cancer surgery and to analyse prognostic factors. To evaluate efficacy and analyse prognostic factors of ^125^I seed implantation for pelvic recurrence after early cervical cancer surgery. A prospective study was conducted on 62 patients who experienced pelvic recurrence after early cervical cancer surgery between August 2005 and September 2015. The 62 patients were treated and assessed in 2 groups (n = 30). All 62 patients were randomized into two groups that received two different treatment regimens: the treatment group (n = 30), which received ^125^I particle implantation therapy, and the control group (n = 32), which received whole-pelvic irradiation using the anteroposterior/posteroanterior field and cisplatin-based concurrent chemoradiation therapy. The efficacy/efficiency of ^125^I seed implantation and prognostic factors were analysed by logistic regression. Overall survival was determined by Kaplan–Meier analysis. Multivariate analysis results were obtained by the Cox proportional hazards regression model. The effective control rates at 1, 3, 6 and 12 months were 76.7%, 80.0%, 83.3%, and 86.7% in the ^125^I particle implantation group. The total effective control rates at 1, 3, 6 and 12 months were 65.6%, 65.5%, 62.5%, and 71.9% in the chemoradiotherapy group. Significant differences were observed between the two groups. The overall survival rates at 1, 2, 3, 4, and 5 years and the median overall were 96.7%, 93.3%, 86.7%, 71.9%, 65.6% and 4.34 years, respectively, in the ^125^I seed implantation group and 81.3%, 71.9%, 62.5%, 56.3%, 53.1% and 3.59 years, respectively, in the control group. There were statistically significant differences in survival rates depending on the diameter of the largest recurrent pelvic tumour (χ^2^ = 6.611, P = 0.010). The multivariate analysis showed that the survival rates were related to the diameter of the largest recurrent pelvic tumour (χ^2^ = 4.538, P = 0.033). ^125^I implantation is an effective, safe, and promising method for the treatment of pelvic recurrence after early cervical cancer surgery. The diameter of the recurrent pelvic tumour was identified as a significant independent prognostic factor in patients who received ^125^I implantation.

## Introduction

Cervical cancer has the fourth highest mortality rate among gynaecologic tumours and is a great threat to women's health worldwide^1^. More than 30% of patients experience recurrence or metastasis after treatment, and locoregional recurrence of the pelvic cavity is the predominant pattern of treatment failure in cervical cancer^2^.

In recent years, because of the prevalence of early screening for cervical cancer, continuous improvements in surgical techniques and advancements in diagnosis and treatment techniques, cervical cancer has been diagnosed earlier and treated in a timely manner, which has substantially reduced the mortality rate^3^. However, the therapeutic results are not satisfactory, and its therapeutic effect and prognosis are affected by many factors^4–7^. Currently, the effects and prognostic factors of recurrent cervical cancer have been the area of intense clinical research, and how to improve the prognosis of patients with recurrent cervical cancer is an urgent problem.

^125^I seeds (half-life: 59.4 days) are permanently implanted in a patient’s body and emit characteristic γ-rays of relatively low photon energy (27.4–28.4 keV) for several months. ^125^I seed implantation in a pelvic mass guided by a high-resolution computed tomography machine can significantly increase the therapeutic dose to the tumour target and reduce the probability of unnecessary damage to surrounding healthy tissues^8, 9^.

^125^I seed implantation via computed tomography (CT)-guided imaging has been widely applied in the local treatment of various kinds of solid tumours, and it has shown clear efficacy^10^. Sixty-two patients who developed a pelvic tumour after cervical cancer surgery were enrolled and subjected to a computer three-dimensional planning system (tps) and a CT and B-scan ultrasonography precision positioning system. The clinical implications of radiation (^125^I) particle implantation for locally recurrent unresectable pelvic tumours were investigated. Pain relief, curative effects, complications and survival times were determined. The results of this study can be used to determine the effect and prognosis of ^125^I particle implantation in patients with pelvic recurrence after cervical cancer surgery and provide a reference for the clinical treatment and prognosis of such patients. To formulate an effective treatment plan, it is of great significance to improve the survival of patients with pelvic recurrence and the long-term curative effect after cervical cancer surgery.

## Results

### Patients and complications

In this study, 62 patients developed recurrence after early cervical cancer surgery, and the average time to recurrence was 70 months (32–100 months). The patient characteristics are given in Table 1.

**Table 1:** Characteristics of 62 patients treated.

Characteristics	^125^I implantation	Radiochemotherapy
No. of patients	30*	32*
Mean age (years)	44.3 (28–65)	46.5 (31–68)
**Pre-operation tumor diameter (cm)**		
< 3	10	13
≥ 3	20	19
**FIGO stage**		
Ia	4	2
Ib	17	17
IIa	9	10
IIb	0	3
**First operation**		
Hysterectomy	2	3
Subtotal hysterectomy	4	6
Radical hysterectomy	24	23
**Histologic type**		
Squamous carcinoma	23	25
Adenocarcinoma	5	5
Other	2	2
**Surgical vaginal margin**		
Positive	3	6
Negative	27	26
**Lymphonodus**		
Positive	5	4
Negative	25	28
**Differentiation**		
Moderately	20	18
High	10	15
**Recurrent pelvic tumor diameter (cm)**		
< 3	8	10
≥ 3	22	22
**Number of recurrent pelvic tumor nodules**		
One	25	26
Two	5	6

*For each characteristic, there is no significant difference in comparison for patients who received ^125^I implantation and for those who underwent radiochemotherapy (*P* > 0.05)**.**

The FIGO stages of the tumours at the first operation were as follows: stage Ia in 6 patients (9.7%), stage Ib in 34 patients (54.8%), stage IIa in 19 patients (30.7%), and stage IIb in 3 patients (4.8%). Among these patients, 58 (93.6%) had squamous cell carcinoma, 10 (16.1%) had adenocarcinoma, and 3 (4.8%) had other tumours. Postoperative pathology showed the following: R1 excision and positive vaginal margins, 9 cases (14.5%); negative vaginal margins, 53 cases (85.5%); moderate or low differentiation, 38 cases (61.3%); high differentiation, 25 cases (40.3%); recurrent tumour volume ≤ 3 cm, 18 cases (29.0%); and recurrent tumour volume > 3 cm, 44 cases (71.0%). Specific data are shown in Table 1.

All patients had mild pain at the puncture site after the operation without special treatment. After 2–3 days, the pain was relieved. Two patients experienced abdominal pain, diarrhoea and a rectal reaction after 3 months. Symptomatic diarrhoea was relieved after treatment.

### Tumour response

The follow-up time ranged from 7.2 to 60 months. Follow-up images showed that the local control rates the ^125^I implantation group after 1 month were as follows: 15 (50.0%), CR; 11 (26.7%), PR; 3 (10.0%), SD; and 1 (3.3%), PD. Those for the radiochemotherapy group were as follows: 12 (37.5%), CR; 11 (34.5%), PR; 4 (12.5%), SD; and 5 (15.6%), PD. After 12 months, the total effective rate in the ^125^I implantation group was 86.7%, which was significantly higher than that of the chemoradiotherapy group (71.9%, P < 0.05). The total effective control rates were 76.7%, 80.0%, 83.3% and 86.7% in the ^125^I particle implantation group, and those at 1, 3, 6 and 12 months in the combined chemoradiotherapy group were 65.6%, 65.5%, 62.5%, and 71.9%. A comparison of the above data revealed a significant difference between the two groups.

**Figure 1:**
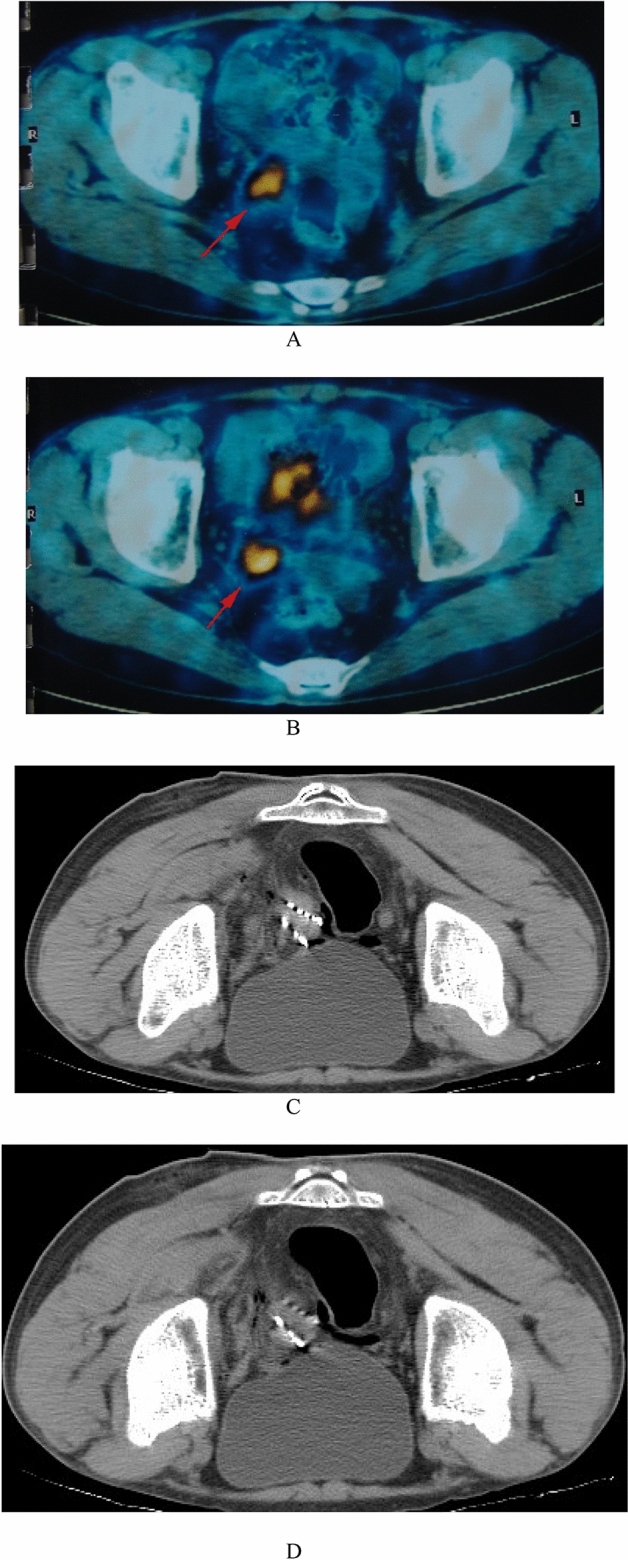
A 35-year-old patient with a tumor of pelvic recurrence after cervical cancer surgery. Tumor dimensions: 20 mm × 2.5 mm × 3.0 mm before treatment. (**A**,**B)** The imaging of PET-CT before multimodality minimally invasive treatment showed abnormal radioactivity concentration in the pelvic. (**C**,**D**) The imaging of CT after the 125I implantation treatment.

**Figure 2:**
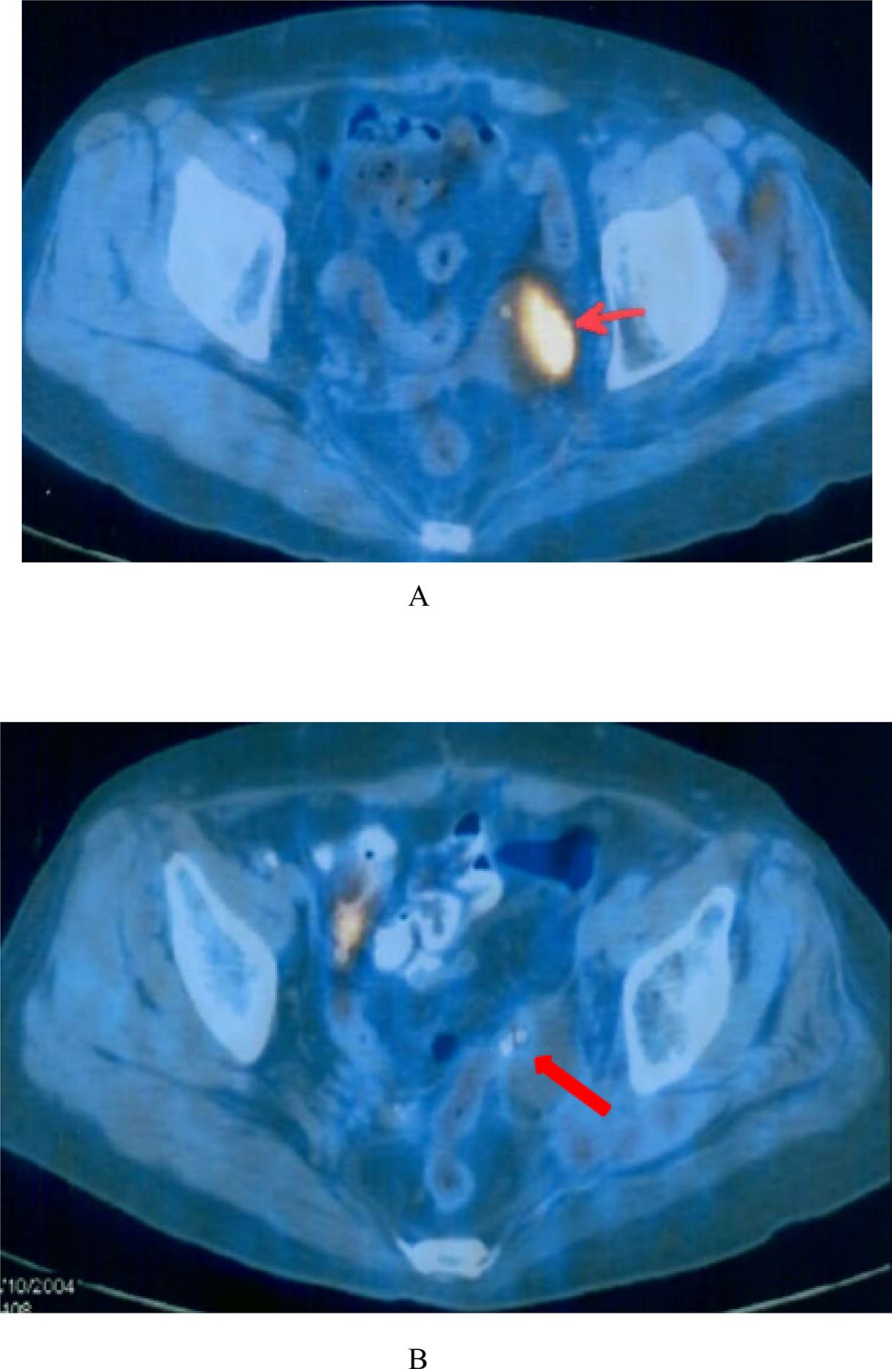
A 47-year-old patient with a tumor of pelvic recurrence after cervical cancer surgery. Tumor dimensions: 20 mm × 1.5 mm × 1.0 mm before treatment. (**A**) The imaging of PET-CT before multimodality minimally invasive treatment showed abnormal radioactivity concentration. (**B**) The imaging of PET-CT after the treatment revealed the absence of abnormal radioactivity concentration and came into a cold lesion.

At ^125^I particle implantation, the 1-year survival rate was 96.7%, the local total effective control rate (RR) was 76.7%, and the progression-free control rate (SD) was 16.7%. In the radiotherapy group, the 12-month survival rate, local total effective control rate and progression-free control rate were 88.3%, 65.6% and 12.5%, respectively. Between the ^125^I implantation group and the radiochemotherapy group, the local total effective control rate was significantly different, but the 1-year survival rate was not. Response details for patients after treatment are given in Tables 2 and 3 and Figs. 1 and 2.

**Table 2:** Comparison of the response rate of patients treated with ^125^I implantation with radiochemotherapy.

Group	No. of patients	Date (m)	Local control efficacy (%)				
			CR**	PR**	NC**	PD**	RR
^125^I implantation	30	1	15 (50.0)*	8 (26.7)	5 (16.7)	2 (6.7)	23 (76.7)*
		3	14 (46.7)*	10 (33.3)	3 (10.0)	3 (10.0)	24 (80.0)*
		6	16 (53.3)*	9 (30.0)	3 (10.0)	2 (6.7)	25 (83.3)*
		12	15 (50.0)*	11 (26.7)	3 (10.0)	1 (3.3)	26 (86.7)*
Radiochemotherapy	32	1	9 (28.1)	12 (37.5)	4 (12.5)	7 (21.9)	21 (65.6)
		3	10 (31.2)	11 (34.3)	5 (15.6)	6 (18.8)	21 (65.5)
		6	10 (31.2)	10 (31.2)	5 (15.6)	7 (21.9)	20 (62.5)
		12	12 (37.5)	11 (34.5)	4 (12.5)	5 (15.6)	23 (71.9)

Data in parentheses are percentages. **CR complete response, *PR* partial response, *NC* no change, *PD* progressive disease, *RR* indicates the percentage of patients with response, i.e. RR = PR + CR. *P < 0.05 versus radiochemotherapy group.

**Table 3:** Comparison of survival of patients treated with ^125^I implantation with conventional radiochemotherapy.

	^125^I implantation	Radiochemotherapy	*P* value
Median survival (months)	4.34 ± 1.19	3.59 ± 1.67	< 0.05
**Overall survival**			
1-year (%)	96.7	88.3	> 0.05
2-year (%)	93.3	71.9	< 0.05
3-year (%)	86.7	62.5	
4-year (%)	71.9	56.3	
5-year (%)	65.6	53.1

### Overall survival

**Figure 3:**
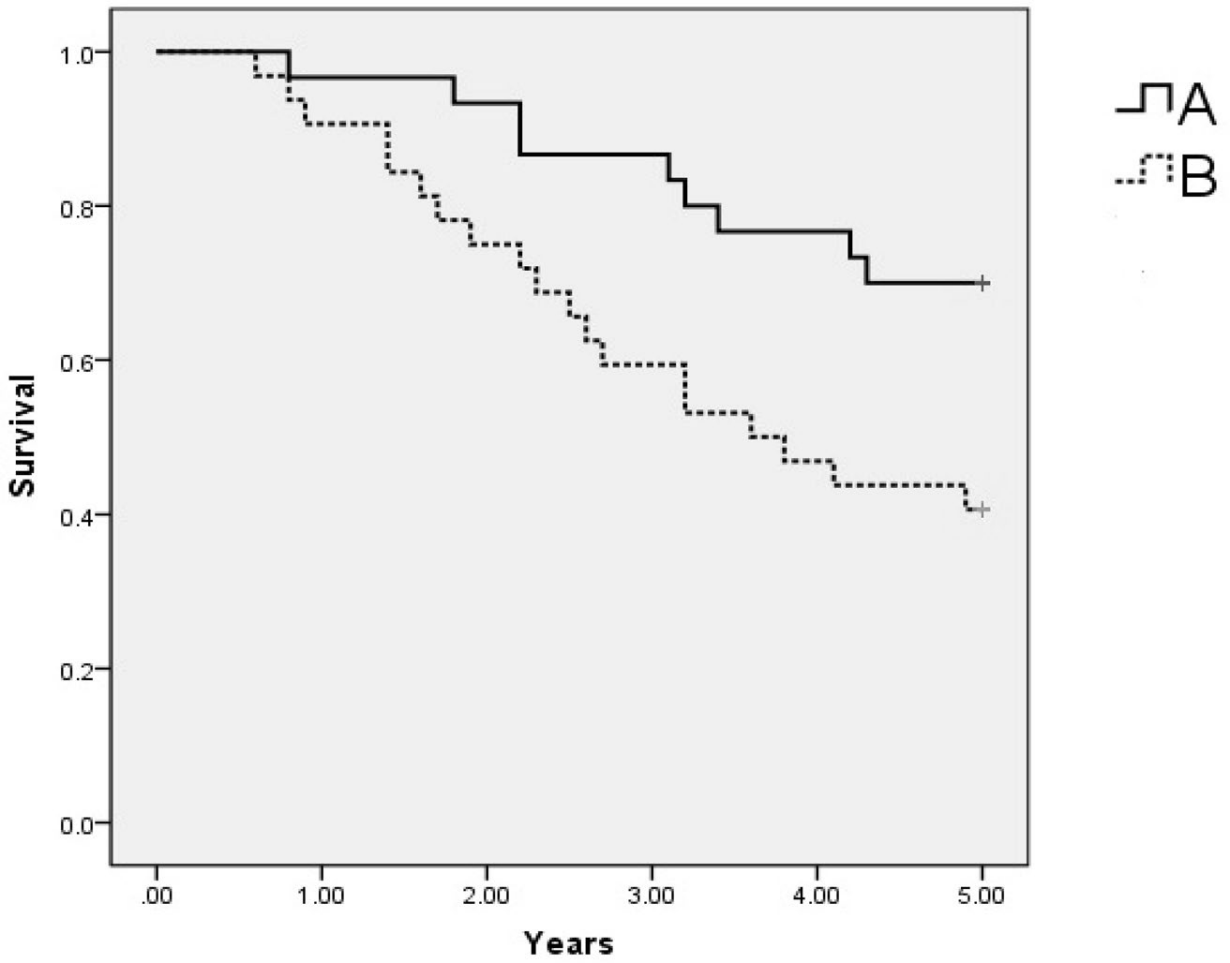
Kaplan–Meier curve shows overall survival rates for patients who received ^125^I implantation (**A**) and for those who underwent radiochemotherapy (**B**).

For the ^125^I implantation group, the 1-, 2-, 3-, 4- and 5-year overall survival rates and the median overall were 96.7%, 93.3%, 86.7%, 71.9%, 65.6% and 4.34 years, respectively, and those for the radiochemotherapy group were 81.3%, 71.9%, 62.5%, 56.3%, 53.1% and 3.59 years, respectively. Mantel–Haenszel tests revealed that the 2-year, 3-year, 4-year, and 5-year overall survival rates for the ^125^I implantation group were significantly better than those for the radiochemotherapy group. The K–M survival curve for the ^125^I implantation group was significantly better than that for the chemoradiotherapy group. The above data are presented in Table 3 and Fig. 3.

### Univariate analysis

The influence of patient- and tumour-related factors on overall survival is shown in Tables 4 and 5. The results showed that the diameter of the largest tumour of the recurrent pelvic tumour was significantly associated with the survival rate (χ^2^ = 6.611, P = 0.010). Statistical results also showed no significant differences in overall survival rates attributable to age at the first operation (χ^2^ = 0.001, P = 0.969), diameter of the largest tumour (χ^2^ = 1.060, P = 0.303), first procedure (χ^2^ = 1.190, P = 0.552), pattern of organization (χ^2^ = 0.388, P = 0.824), FIGO stage (χ^2^ = 0.025, P = 0.988), vaginal incision (χ^2^ = 0.002, P = 0.962), lymph node invasion (χ^2^ = 0.389, P = 0.533), degree of differentiation (χ^2^ = 0.645, P = 0.422), or ^125^I particle usage (χ^2^ = 0.615, P = 0.433).

**Table 4:** Results of univariate Kaplan–Meier survival analysis with Log-Rank test for various prognostic factors.

Factor	No. of patients (%)	Overall survival (%)	χ^2^	P value
**Age (years)**				
< 40	10 (33.3)	70.0	0.001	0.969
≥ 40	20 (66.7)	70.0		
**Pre-operation diameter of largest tumor (cm)**				
< 3	14 (46.7)	78.6	1.060	0.303
≥ 3	16 (53.3)	62.5		
**First** **operation**				
Hysterectomy	2 (6.7)	50	1.190	0.552
Subtotal hysterectomy	5 (16.7)	60		
Radical hysterectomy	23 (76.7)	73.9		
**Histologic type**				
Squamous carcinoma	26 (86.7)	69.2	0.388	0.824
Adenocarcinoma	3 (10.0)	66.7		
Other	1 (3.4)	100		
**FIGO stage**				
IA	4 (13.3)	75.0	0.025	0.988
IB	17(56.7)	70.6		
IIA	9(30.0)	66.7		
**Surgical vaginal margin**				
Positive	3 (10.0)	66.7	0.002	0.962
Negative	27 (90.0)	70.4		
**Lymphonodus**				
Positive	5 (16.7)	60.0	0.389	0.533
Negative	25 (83.3)	72.0		
**Differentiation**				
Moderately	20 (66.7)	65.0	0.645	0.422
High	10 (33.3)	80.0		
**Recurrent pelvic tumor diameter(cm)**				
< 3	8 (26.7)	37.5	6.611	0.010
≥ 3	22 (73.3)	81.8		
**Number of I-125 particles used**				
< 20	10 (33.3)	60.0	0.615	0.433
≥ 20	20 (66.7)	75.0

**Table 5:** Multivariate analysis of prognostic factors with Cox proportional hazards model.

Variable	β value	Standard error	Wald χ^2^ value	P value	RR	95% confidence interval	
Age (years)	− 1.519	1.102	1.903	0.168	0.219	0.025	1.896
Pre-operation diameter of largest tumor	0.398	1.141	0.122	0.727	1.489	0.159	13.936
First operation	1.418	0.855	2.751	0.097	4.131	0.773	22.079
histologic type	0.017	1.096	0.000	0.988	1.017	0.119	8.717
FIGO stage	0.161	0.754	0.046	0.831	1.175	0.268	5.146
surgical vaginal margin	− 1.622	1.543	1.105	0.293	0.197	0.010	4.065
Differentiation	0.086	1.279	0.005	0.946	1.090	0.089	13.365
Maximum diameter of recurrent pelvic tumor after surgery	− 2.011	0.944	4.538	0.033	0.134	0.021	0.851
Number of I-125 particles used to treat recurrent pelvic tumors	− 0.710	1.153	0.379	0.538	0.491	0.051	4.712

### Multivariate analysis

**Figure 4:**
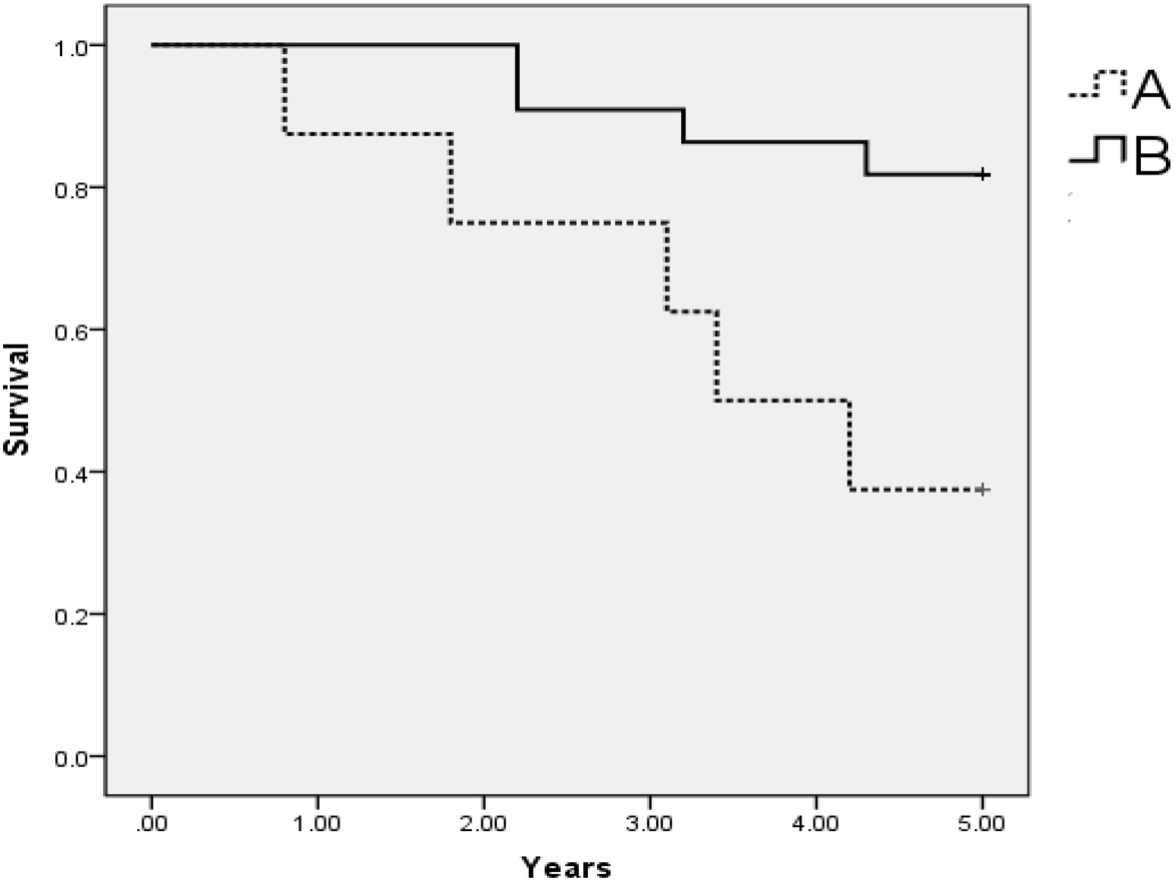
Graph shows 5-year cumulative survival rate after ^125^I implantation therapy, compared between patients with diameter of largest tumor ≥ 3 cm (**A**) and < 3 cm (**B**) in recurrent pelvic tumor after cervical cancer surgery. The survival rate of patients with diameter of largest tumor < 3 cm was significantly higher than that of patients with diameter of largest tumor ≥ 3 cm.

Multivariate analysis was performed by using the variables deemed significant in the univariate analysis as covariates. The multivariate analysis (Table 5; Fig. 4) showed that the survival rates were related to the diameter of the largest tumour of the recurrent pelvic tumour (χ^2^ = 4.538, P = 0.033).

## Discussion

Permanently implanted ^125^I particle tissue is a close-range radiotherapy method in which radioactive particles are implanted into tumours or invaded tissues, killing tumour cells in the target area by continuously releasing γ rays. This method has the biophysical characteristics of a high dose, high conformability and a steep decrease in the dose to surrounding tissue. The pathological damage is relatively minimal, and the damage range is limited to 5 mm from the source^8^. By adjusting the spacing and activity of the particle source, the dose to the tumour target area can be increased exponentially, which can ensure that the target area is exposed to high intensity and protect the surrounding normal organs to the greatest extent^9^. At the same time, the radiation-related toxic side effects do not increase^10^. Particle implantation has been increasingly considered for prostate cancer. With the wide application of radioactive ^125^I particles, there are many reports of permanent inter-tissue irradiation in the treatment of diverse solid tumours, and the particles have achieved a good curative effect^11^. Thus, they can be used as a supplementary treatment for recurrent pelvic malignant tumours.

Permanent inter-organizational implantation of radioactive ^125^I particles is a safe and effective treatment for brachytherapy, and it has the advantages of minimal invasiveness, a high local dose, small injury to surrounding normal tissue and a low incidence of complications. Our centre uses CT-guided radioactive particle implantation to treat recurrent cervical cancer, but because of the complex anatomical structure and bone structure of the pelvic cavity, it is difficult to maintain a parallel arrangement of ^125^I particles^12^. With the emergence of 3D printing technology and 3D⁃PNCT combined with CT guidance technology, this problem has been solved^13^.

This study describes the use of ^125^I particle implantation for pelvic recurrence after early cervical cancer surgery. Clinical and pathological characteristics were used to perform a detailed statistical analysis to determine the efficacy of radiotherapy on pelvic recurrent tumours and prognostic factors. The results of this study can provide a reference for the clinical treatment and prognosis of patients with pelvic recurrence after cervical cancer surgery to formulate more effective treatment plans. It is very important to improve the survival of patients with pelvic recurrence after cervical cancer surgery and to improve the long-term curative effect^14^.

Many studies have shown that the size of the recurrent tumour is an important factor affecting the curative effect and prognosis of patients with pelvic recurrence after cervical cancer surgery. Hisao lto and others reported 90 cases of vaginal stump recurrence after cervical cancer surgery according to the size of the recurrent tumour (divided into three groups). The 5-year survival rate was 0% in zero patients with a recurrent tumour ≥ 3 cm, 56% in 18 patients with a recurrent tumour < 3 cm, and 87% in 37 patients with non-obvious tumour nodules. The difference was statistically significant (P < 0.05). The results showed that the efficacy of the non-obvious lesion group was better than that of the other two groups, the size of the recurrent tumour was significantly correlated with prognosis, and the size of the recurrent tumour was an important prognostic factor (P = 0.01)^15^. ^125^I seed implantation can be used to treat pelvic recurrence from cervical cancer. Qu et al.^16^ evaluated the efficacy and dosimetry of ^125^I seed implantation for pelvic recurrence from cervical cancer by examining 36 patients between July 2005 and October 2015. Nineteen (52.7%) achieved partial response (PR), 13 (36.1%) had no change (NC), and 4 (11.2%) developed progressive disease (PD). The total effective rate (CR + PR) was 88.9%, and the progression-free survival (PFS) time of 4 patients was no longer than 12 months. The median survival time was 7.5 months. The 1- and 2-year local progression-free survival rates were 34.9% and 20%, respectively^16^.

In this study, after ^125^I seed treatment, the 5-year overall survival rate of patients with a recurrent tumour ≥ 3 cm was 37.5% and that of patients with a recurrent tumour ≤ 3 cm was 81.8%. The 5-year survival rates are shown in Table 4. Single-factor analysis in this study showed that the diameter of the locally recurrent pelvic tumours after cervical cancer surgery influences ^125^I particle therapy outcomes. Multiple-factor analysis showed that the size of the recurrent tumour was statistically significant for prognosis (P = 0.033). These findings suggest that the size of the recurrent tumour is not only a related factor affecting the therapeutic effect and prognosis of pelvic recurrence after cervical cancer surgery but also the only independent factor affecting its therapeutic effect and prognosis. Therefore, the larger the recurrent tumour is, the worse the prognosis, which may be due to the large size of the tumour. Short-range treatment cannot be used to reach the maximum total tumour dose within the limit of radiotherapy toxicity. Moreover, if the tumour is large, there are many oxygen-deficient cells, the sensitivity to radiotherapy decreases, and metastasis easily occurs. Therefore, for patients with large recurrent tumours after radiotherapy and/or residual tumours, multi-modal treatment, such as combined chemotherapy, can be considered, which may improve the curative effect^17^.

There are still several problems in the treatment of recurrent pelvic tumours after cervical cancer surgery. Although previous studies used full pelvic irradiation, recurrence still occurred after treatment, and whether the dose of radiotherapy needs to be increased and whether chemotherapy should be maintained after synchronous radiotherapy and chemotherapy are still controversial. Siriwan Tangjitgamol and others reported that after concurrent chemoradiotherapy, the survival of patients with locally advanced cervical cancer was improved by maintenance chemotherapy. However, there were several potential controversies in this trial, including an inappropriate statistical design, and there are no related reports on advanced toxicity associated with intensive chemotherapy. Therefore, the role of maintaining chemotherapy remains to be further studied^18^.

In our study, individualized brachytherapy plans were established before treatment based on different recurrence sites and tumour sizes can improve survival. Given that a higher radiotherapy dose for recurrent pelvic wall tumours is an effective measure to improve the local control rate and prognosis of patients with recurrent pelvic tumours, it is particularly important for clinicians to develop a reasonable and scientific individualized treatment for pelvic recurrent tumours after cervical cancer surgery^19^. However, our study still had limitations. First, we did not further study the mechanism of treatment differences. Second, the competitive risks of the Fine and Gray type were not assessed in this study, which may have resulted in competitive risk bias, but the direct cause of patient death in this study was cancer, this study is still valuable and needed for future studies. We also plan to perform a further study by combining this brachytherapy with systemic radiochemotherapy.

## Methods

### Patients

Between August 2005 and September 2015, a total of 62 patients who were diagnosed with pelvic recurrence after cervical cancer surgery and who were admitted to two individual hospitals working collaboratively were included in the current study. All participating patients were approved by the Ethical Committee of GuangZhou Red Cross Hospital. Before treatment initiation, all subjects were fully aware of the potential risks and provided written informed consent. Our study was performed in accordance with all international, national, and institutional policies regarding research studies involving human subjects.

Patients who developed a pelvic tumour after early cervical cancer surgery were randomized into two groups: the experimental group, which included 30 patients who received ^125^I particle implantation, and the radiation and chemotherapy group, which included 32 patients who received pelvic irradiation at the prescribed dose of ≤ 46 Gy and a dual regimen consisting of platinum concurrent chemotherapy. Randomization was achieved by using computer-generated random numbers. Detailed information on each group is presented in Table 1. There was no statistically significant difference between the two groups (P > 0.05). All patients had been examined by surgeons and radiation oncologists and were deemed unsuitable for resurgery. The five patients in the treatment group with positive lymph nodes were deemed unsuitable for EBRT, and some patients refused to undergo surgery and EBRT.

### Eligibility criteria

The strictly enforced entry criteria were as follows: (1) patient KPS ≥ 90; (2) tumour recurrence was confirmed by pathology and immunohistochemistry or had at least two of the following positive results: (scc-ag) level rise, CT, MRI and PET-CT indicated recurrence; and (3) the leucocyte count was above 3.5 × 10^9^/L before surgery or could be elevated to this level after supportive treatment.

### Exclusion criteria

The strictly enforced exclusion criteria were as follows: (1) a history of postoperative adjuvant therapy; (2) a surgical history of early cervical cancer or received preoperative radiotherapy and postoperative radiotherapy; (3) para-aortic lymph node recurrence or distant metastasis; (4) only palliative treatment was given after recurrence; and (5) patients with severe cardiovascular and cerebrovascular diseases, liver and kidney diseases and other systemic diseases.

### Instruments

A Picker CT-Twin Flash scanner was used to image the tumour at 120 kV and 275 mA with a slice width of 5 mm. The treatment planning system (TPS) and radioactive seed treatment planning system (BT-RSI) were developed by Yuanbo (Beijing, China)^9^. The main instruments required for seed implantation included a turn-table implantation gun, 18-G implantation needles and ^125^I seeds, which were the most important (i.e., the therapeutic part). ^125^I produces gamma rays (95% of 28 keV, 5% of 35 keV), has a half-life of 59.6 days, a half-value thickness of 0.025 mm of lead, a penetration depth of 17 mm, and an initial rate of 7 cGy/h^20, 21^. Each seed has a diameter of 0.8 mm and a length of 4.5 mm, and the titanium capsule wall has a thickness of 0.05 mm. The ^125^I seeds are made from ^125^I absorbent silver rods and are encapsulated in a laser-welded titanium envelope. All the ^125^I seeds (6711/BT-125I) were mailed to our hospital in a type-A package that had passed leak detection and activity series tests.

### ^125^I seed implantation planning

**Figure 5:**
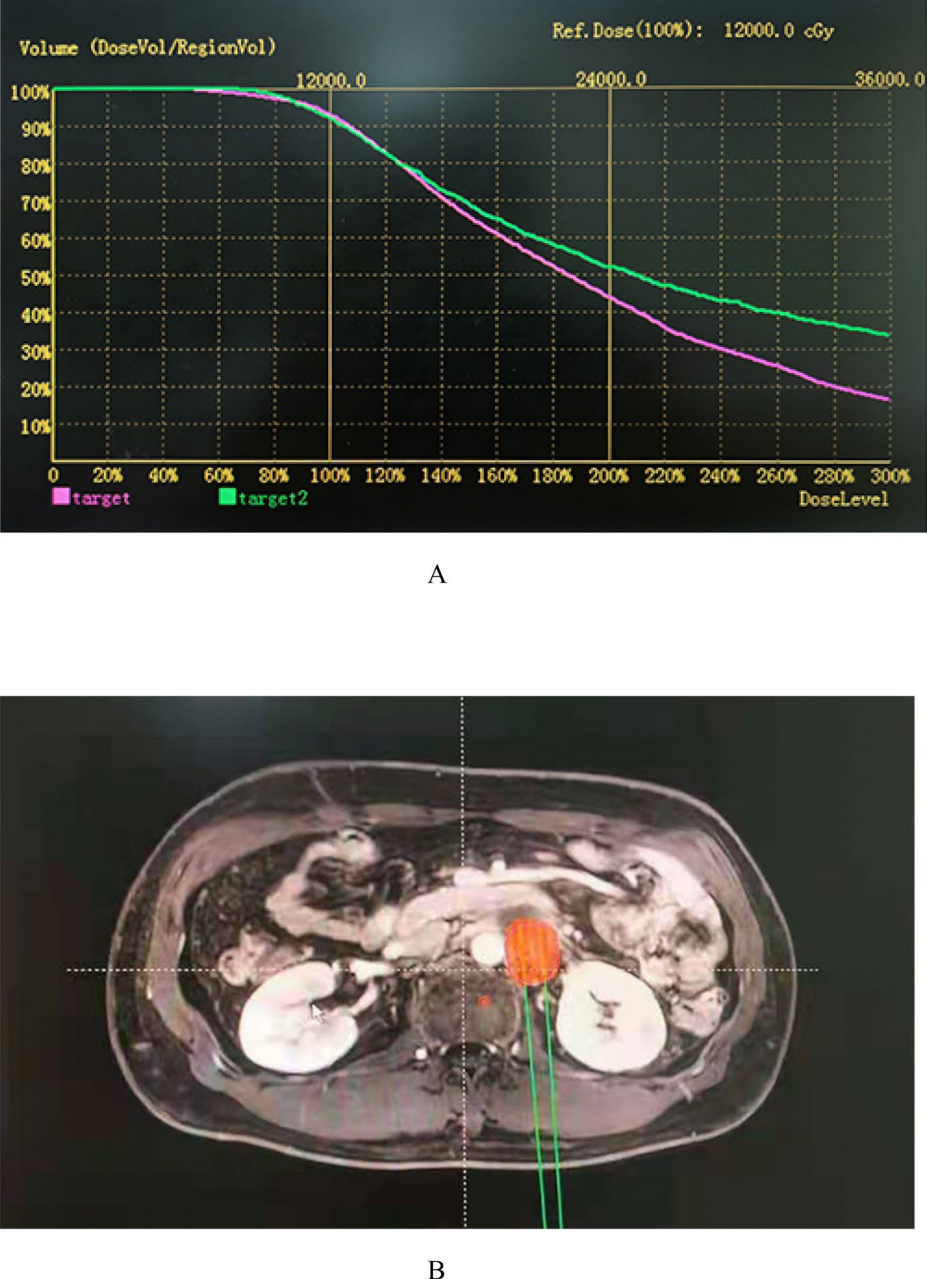
A 50-year-old patient with a tumor of common iliac lymph node recurrence after cervical cancer surgery. (**A**) Radiotherapy specialists design dose-volume histograms on a treatment planning system based on the patient's tumor profile. (**B**) The location and dose of ^125^I seeds were calculated using two-dimensional images of the tumor from the treatment planning system.

A detailed analysis of tumour volume was performed 1–2 weeks before seed implantation using a 5 mm thick CT scan. On each cross-sectional image, the radiation oncologist outlined the gross tumour volume (GTV) and the risk area for subclinical disease. The planned target volume (PTV) included the entire GTV and a boundary of 0.5–1.0 cm^22^. The dose was prescribed as the minimum peripheral dose (MPD), which included the PTV. The median MPD was 130 Gy (range 90–160 Gy)^23^. The distribution and MPD of ^125^I seeds were calculated by using a computer processing planning system (RT-RSI, Beijing Atom and High Technique Industries Inc., Beijing, China)^24^. An example is shown in Fig. 5.

### CT-guided ^125^I implantation and radiation protection

The patients fasted for 1.0–3.0 h and were given sedatives and local anaesthesia before the procedure. Based on the established treatment plan, a 3-mm incision was made on the skin, and a seed implantation applicator was inserted into each tumour under the guidance of CT at a distance. Care was taken not to puncture large blood vessels or vital organs. For tumours less than or equal to 1.0 cm in size, interstitial planar (surface) implants were used. After the procedure, the catheters were retracted, and incisions were bound and compressed^24^.

A surgeon performed ^125^I implantation in a specially designated operating room. During the operation, the medical personnel wore lead gloves, hats, ambi-necks, and other protective clothing. After the procedure, a special technician detected the ray dosage in the surroundings in real time to detect any missing seeds or other problems.

### Chemotherapy

Thirty-two patients received cisplatin (70 mg/m^2^ per surface area, day 1 of each cycle) combined with 5-fluorouracil (1000 mg/m^2^ per surface area, days 2–5 of each cycle), which was repeated every 4 weeks for a total of 4–6 cycles. Routine blood tests were performed 1–2 times a week during treatment, and other haematologic tests, including liver and kidney function tests, were reviewed before and after each chemotherapy cycle.

### Evaluation criteria

The effectiveness of ^125^I seed implantation was based on the Response Evaluation Criteria in Solid Tumors (RECIST 1.0). The RECIST 1.0 guideline is currently recognized as the most commonly used guideline. This guideline, updated in 2000, is an anatomic-based rather than a functional evaluation system. (1) Complete response (CR) was defined as follows: disappearance of all target lesions. (2) Partial response (PR) was defined as follows: a decrease of at least 30% in the sum of the LD of the target lesions, taking as a reference the baseline sum of the longest diameter. (3) Stable disease (SD) was defined as follows: neither sufficient shrinkage to qualify for PR nor a sufficient increase to qualify for PD, taking as a reference the smallest sum of the longest diameter since the treatment began. (4) Progressive disease (PD) was defined as follows: at least a 20% increase in the sum of the longest diameter of the target lesions, taking as a reference the smallest sum of the longest diameter recorded since the treatment began or the appearance of one or more new lesions. The total treatment response rate (RR) was calculated as RR = (patients who achieved CR + patients who achieved PR)/number of patients^25^.

### Follow-up

Vital signs were monitored for 24 h after implantation. All changes in symptoms were recorded. All patients were hospitalized for at least 3 days for close observations of postoperative adverse events. The average follow-up time for reexaminations and evaluations was 2–3 months. Imaging examinations, such as CT or MRI, were used to evaluate lesion recurrence. MRI or CT results pre- and posttreatment were compared in detail in all patients.

### Statistical analysis

All values are expressed as the mean ± standard deviation. The overall survival curve generated by the Kaplan–Meier method using SPSS 22.0 is shown in Fig. 3, where deaths from all causes were classified as events. A multivariate analysis of prognostic factors (by Cox proportional hazards regression) was performed to calculate the hazard ratios and confidence intervals. T tests were used to evaluate the significance of differences. Models also included age (< 40 years and ≥ 40 years), preoperation diameter of the largest tumour (< 3 cm and ≥ 3 cm), first operation, histologic type (squamous carcinoma, adenocarcinoma, other), FIGO stage (IA, IB, IIA), surgical vaginal margin (positive, negative), differentiation (moderate, high), maximum diameter of the recurrent pelvic tumour after surgery(< 3 cm and ≥ 3 cm), and number of I-125 particles used (< 20 grains and ≥ 20 grains). P values less than 0.05 were considered significant.

The survival time was calculated from the date of diagnosis to the date of death or the last follow-up. Local recurrence was defined as tumour progression within the implanted area or surrounding regions, as seen on CT. Local recurrence and distant metastasis were scored until patient death and censored thereafter.
